# Exosomal arrow (Arr)/lipoprotein receptor protein 6 (LRP6) in *Drosophila melanogaster* increases the extracellular level of Sol narae (Sona) in a Wnt-independent manner

**DOI:** 10.1038/s41419-020-02850-x

**Published:** 2020-11-03

**Authors:** Jeong-Hoon Han, Yeon Kim, Kyung-Ok Cho

**Affiliations:** Department of Biological Sciences, Korea Advanced Institute of Science and Technology, 291 Daehak-Ro, Yuseong-Gu, Daejeon Korea

**Keywords:** Extracellular signalling molecules, Differentiation

## Abstract

Wg/Wnt as a signaling protein binds to Frizzled (Fz) and Arrow (Arr), two Wg co-receptors essential for Wg signaling for cell proliferation, differentiation, and cell survival. Arr has a long extracellular region, a single transmembrane domain and an intracellular region. Here, we report that a new *arr*^*m7*^ mutant is identified in a genetic screen as a suppressor of lethality induced by overexpression of Sol narae (Sona), a secreted metalloprotease in ADAMTS family involved in Wg signaling. *arr*^*m7*^ allele has a premature stop codon, which encodes Arr^m7^ protein missing the intracellular region. *arr*^*m7*^ clones show cell death phenotype and overexpression of Arr^m7^ protein also induces cell death. Levels of extracellular Sona were decreased in both *arr*^*m7*^ and *arr*^*2*^ null clones, demonstrating that Arr increases the level of extracellular Sona. Indeed, Arr but not Arr^m7^, increased levels of Sona in cytoplasm and exosome fraction by inhibiting the lysosomal degradation pathway. Interestingly, Arr itself was identified in the exosome fraction, demonstrating that Arr is secreted to extracellular space. When Sona-expressing S2 cells were treated with exosomal Arr, the extracellular level of active Sona was increased. These results show that exosomal Arr dictates Sona-expressing cells to increase the level of extracellular Sona. This new function of Arr occurred in the absence of Wg because S2 cells do not express Wg. We propose that Arr plays two distinct roles, one as an exosomal protein to increase the level of extracellular Sona in a Wnt-independent manner and the other as a Wg co-receptor in a Wnt-dependent manner.

## Introduction

Wnt signaling is an important pathway conserved in all metazoans for cell proliferation, cell survival, and differentiation^1–4^. Its malfunction causes developmental disorders, metabolic diseases, cancer development, and metastasis^5–8^. Wnt signaling is initiated by the binding of extracellular Wnt to the receptor complex containing Frizzled (Fz) as a main receptor and Arrow (Arr)/Lipoprotein receptor protein 6 (LRP6) as a co-receptor. Arr/LRP6 is a type I transmembrane protein that belongs to low density LRP family^9^. Interaction between Wnt and co-receptors results in a cascade of signaling transduction that leads to gene expression^10,11^.

**Fig. 1:**
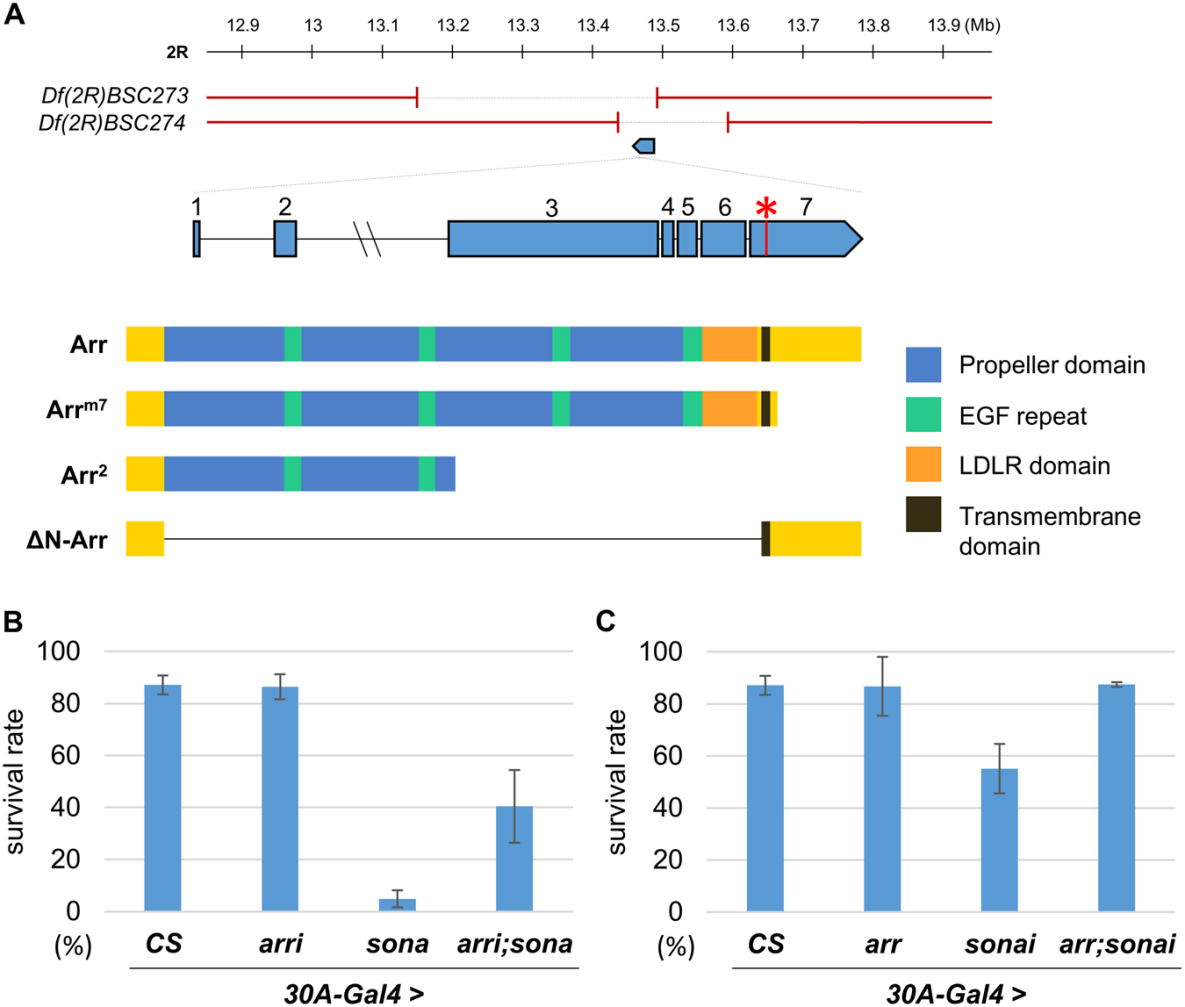
Mapping of arr^*m7*^ suppressor and genetic interaction between *arr* and *sona*. **a** Genetic mapping of *arr*^*m7*^. The *arr* gene is mapped to the region deleted in both deficiencies, *Df(2R)BSC273* and *Df(2R)BSC274*. The exons and introns of the *arr* gene are shown with the nonsense mutation site (G to A) marked with an asterisk. Wild-type and deletion forms of Arr are shown with domain structures. ΔN-Arr protein lacks the extracellular domain spanning 88–1450th amino acid residues while Arr^m7^ protein lacks the intracellular domain. **b**, **c** Experiments were carried out three times and flies were cultured at 25 °C. Adult progenies from crosses between *30A-Gal4*/*CyO* and *Canton S* (*CS*) (*n* = 137 + 92 + 233 from 3 experiments), *UAS-arr RNAi* (*n* = 85 + 129 + 83 from 3 experiments), *-sona* (*n* = 103 + 166 + 154 from 3 experiments), *-arr RNAi; -sona* (*n* = 97 + 121 + 219 from 3 experiments) were obtained (**b**). Adult progenies from crosses between *30A-Gal4*/*CyO* and *UAS*-*arr-HA* (*n* = 86 + 81 + 102 from 3 experiments), *-sona RNAi* (*n* = 89 + 57 + 137 from 3 experiments), *-arr-HA; -sona RNAi* (*n* = 31 + 52 + 53 from 3 experiments) were obtained (**c**). Data are presented as mean ± S.D.

Arr/LRP6 consists of a long extracellular region containing four β-propeller/epidermal growth factor repeats (E1–4) crucial for Wnt binding^12,13^, and three LDL repeats (LDLR) essential for dimerization of Arr/LRP6 proteins^14^ (Fig. 1). LRP6 without E1–4 activates Wnt signaling without Wnt, establishing that E1–4 negatively regulates Wnt signaling^9^. On the contrary, dimerization of LRP6 via LDLR domain is prerequisite for activating Wnt signaling^14^. Upon Wnt binding, the intracellular domain of LRP6 becomes phosphorylated, which eventually leads to stabilization of Armadillo (Arm)/β-catenin for the canonical Wnt signaling^9,15^.

We identified *arr*^*m7*^ as a suppressor of Sona-induced lethality in a genetic screen^16^. Other *sona* suppressors include *wntless* (*wls*), *pou domain motif 3* (*pdm3*), and *archipelago* (*ago*), which all show genetic interaction with *wingless* (*wg*)^16–18^. Sona is a member of a disintegrin and metalloprotease with thrombospondin motif (ADAMTS) family that processes extracellular components. Sona promotes cell survival cell autonomously and increases cell proliferation in a paracrine manner^19^. Furthermore, extracellular Sona cleaves Wg and generates Wg-CTD that is another active form of Wg^17,20^. Wg-CTD is less stable than full-length Wg, and is more specialized for cell proliferation than differentiation^20^. Taken together, *sona* and *sona* suppressors are linked to Wg signaling.

Wg and Sona are secreted to extracellular space by both conventional Golgi transport and exosome secretion pathway^20^. Exosomes are extracellular vesicles (EVs) produced in the endosomal compartment^21^. Intraluminal vesicles inside of multivesicular body (MVB) are released as exosomes when MVB is fused to the plasma membrane. Exosomes contain mixed populations of EVs that deliver proteins, nucleic acids such as tRNAs and noncoding RNAs, and metabolites to neighboring cells^22–26^. Multiple Wnt signaling components are also secreted by the exosomal secretion pathway. For instance, Wls-containing exosomes deliver Wg from presynaptic neuron to postsynaptic muscle for development of larval neuromuscular junction^27^. β-catenin is packaged into exosomes and secreted, which results in downregulation of Wnt signaling^28^. Given that Sona is also involved in Wg signaling and is secreted by exosomal secretion pathway, Wnt signaling components may be commonly present in exosomes.

We focused on the biochemical relationship between Arr and Sona to understand the underlying mechanism of their genetic interaction. We found that the level of extracellular Sona is decreased in *arr* clones in wing discs. Consistent with this clonal analysis, overexpressed Arr stabilized Sona by inhibiting the lysosomal degradation pathway, which leads to increased levels of both intracellular and extracellular Sona in fly Schneider 2 (S2) cells. Interestingly, Arr was present in the exosome fraction, raising a possibility that extracellular Arr is also involved in stabilization of Sona. Indeed, more exosomal Sona was secreted from the Sona-expressing S2 cells cultured in the presence of exosomal Arr. This report shows for the first time that exosomal Arr stabilizes intracellular Sona and then increases the level of exosomal Sona.

## Materials and methods

### Fly lines

*arr*^*m7*^ mutant was derived from *m7 sona* suppressor as described^16^. *UAS-arr*^*m7*^*-HA*, *FRT42D arr*^*m7*^, and *FRT42D arr*^*2*^ are generated with *arr*^*m7*^ and BDSC #3287 for this study. *UAS-sona* lines are generated in our laboratory^17^. *UAS-arr-HA* is a generous gift from Konrad Basler. Other lines are obtained from BDSC and VDRC stock centers.

### DNA constructs

To generate *pUAST-sona-Myc*, the *sona* cDNA^17^ was inserted into *pUAST-Myc* vector. *pUAST-GFP-wg* was generated by recombining *pUAST* vector and *GFP-wg* as described^20^. *pUAST-arr* and *pUAST-arr-HA* were generated by cloning *arr* cDNA clone from DGRC into *pUAST* and *pUAST-HA* vectors, respectively. *ΔN-arr-HA* was generated by deleting a DNA region encoding 88–1450th amino acid residues from *arr-HA*.

### Cell culture, transfection, and preparation of various fractions

S2 and S2 R+ cell lines (DGRC) were cultured in M3 media (Sigma-Aldrich S8398) containing 10% insect medium supplement (Sigma-Aldrich I7267) at 25 °C. Media for S2 R+ cell lines contained additional yeast extract and bactopeptone. Transfection was performed with effectene transfection reagent (Qiagen). For P100 preparation, conditioned media were sequentially centrifuged at 240×*g*, 2000×*g*, 10,000×*g* and 100,000×*g*^29^. After 100,000×*g* centrifugation, supernatant fraction (SN_Δ_) was concentrated with a filter column. The obtained P100 fraction contains EVs including exosomes, was washed with PBS, and then pelleted again by additional centrifugation at 100,000×*g*.

### Immunocytochemistry

Sona-Pro and Sona-C antibodies are used to detect Sona protein as described^17^. The Arr antibody is a generous gift from Suzanne Eaton. For immunocytochemistry, fly larvae were dissected and fixed with PLP solution (2% paraformaldehyde, 0.1 M lysine, 0.25% sodium M-periodate) as described^30^. Fixed wing discs were blocked in block buffer (50 mM Tris pH 6.8, 150 mM NaCl, 0.2% TritonX-100, 5 mg/ml bovine serum albumin) for 2–6 h at 4 °C and washed in wash buffer (0.2% TritonX-100 in PBS). Antibodies were diluted in wash buffer and incubated overnight at 4 °C or 2 h at room temperature. After washing several times, samples were treated with DAPI and mounted with a Vectashield mounting medium. Sample images were captured with Zeiss LSM laser scanning confocal microscope and processed by Adobe Photoshop. Sona-Pro (rabbit, 1:200), Sona-C (mouse, 1:100), HA (Roche 3F10, rat, 1:200), Arr (guinea pig, 1:150), cleaved caspase 3 (Cell signaling Asp175, rabbit, 1:100), Wg (DSHB 4D4, mouse, 1:500), and β-gal (Abcam ab9361, chicken, 1:100) were used.

For extracellular staining, larvae were dissected in ice-cold M3 medium and incubated in M3 medium containing primary antibody for 2 h at 4 °C as described^31^. Then, wing discs were washed with M3 and PBS twice each, and fixed in 4% paraformaldehyde in PBS for 50 min. Samples were then processed by the same way as described above but buffers did not contain any detergent. The antibodies were used at ten times higher concentration for extracellular staining than intracellular staining.

### Western analysis and co-immunoprecipitation

Western analysis was performed as described with Thermo Scientific West Pico Plus Chemiluminescent Substrate for ECL system^17^. Sona-Pro (rabbit, 1:5000), HA (Roche 3F10, rat, 1:2000), Arr (guinea pig, 1:3000), Wg (DSHB 4D4, mouse, 1:500) and α-tubulin (Sigma-Aldrich T9026, mouse, 1:5000–10,000) were used.

For co-IP, protein G agarose beads (Merck Millipore) was incubated in primary antibody containing lysis buffer (20 mM HEPES, 150 mM NaCl, 2 mM DTT, 5 mM EDTA, 5 mM EGTA, 0.2% Triton X-100, 10% glycerol and Roche complete protease inhibitor cocktail) for 2 h at room temperature. Pelleted cells were incubated in the lysis buffer on ice for 30 min and cell lysates were precleared with beads for 30 min. This precleared cell lysate was incubated with antibody-conjugated beads overnight at 4 °C. Then, beads were washed with the lysis buffer several times and used for western analysis.

## Results

### arr^m7^ is identified as a sona suppressor

We previously identified suppressors that overcome lethality induced by overexpressed Sona in a genetic screen^17^. A suppressor *m7* had two lethal sites and one of them was present in *pdm3* as reported^16^. Pdm3 induces *wg* transcription and is essential for the development of neuromuscular junctions^16^. The other lethal site was mapped to the region deleted in two deficiencies, *Df(2R)BSC273* and *Df(2R)BSC274* (Fig. 1a). Complementation test and whole-genome sequencing determined that the second lethal site is located in the *arr* gene, and was named *arr*^*m7*^. *arr*^*m7*^ had a nonsense mutation in the seventh exon of the *arr* gene, which truncates 191 amino acid residues of the intracellular region from 1678 amino acid residues of Arr (Fig. 1a).

### Arr functions downstream to Sona

To confirm genetic relationship between *arr* and *sona*, we co-expressed *UAS-arr RNAi* and *UAS-sona* and checked whether lethality by *sona* overexpression is suppressed by knockdown of *arr* (Fig. 1b). Survival rate of *30A-Gal4* > *UAS-arr RNAi* (*30A* > *arr RNAi*) flies was 86%, similar to that of *30A-Gal4/+* control flies. On the other hand, the survival rate of *30A* > *sona* flies was only 5% and that of *30A* > *GFP; sona* was 6%, and both flies showed late pupal lethality (Fig. 1b and S1). Co-expression of *arr RNAi* and *sona* increased the survival rate to 40% (Fig. 1b). Furthermore, all *30A* > *sona* flies died in a few hours after eclosion whereas 50% of *30A* > *arr RNAi; sona* files were normal and fertile. Therefore, the lethal phenotype induced by gain of *sona* was significantly rescued by knockdown of *arr*.

We then tested whether gain of *arr* can rescue the lethality induced by knockdown of *sona*. Survival rates of *30A* > *arr-HA* flies and *30A-Gal4/+* control flies were both 87% (Fig. 1c). The survival rate of *30A* > *sona RNAi* flies was 55% whereas that of *30A* > *sona RNAi; arr-HA* flies was 87% (Fig. 1c). Therefore, the lethal phenotype induced by knockdown of *sona* was fully rescued by gain of *arr*. Since the level of Arr affected phenotypes induced by either knockdown or gain of Sona, we concluded that *arr* functions downstream to *sona*.

### Arr^m7^ is dominant negative

**Fig. 2:**
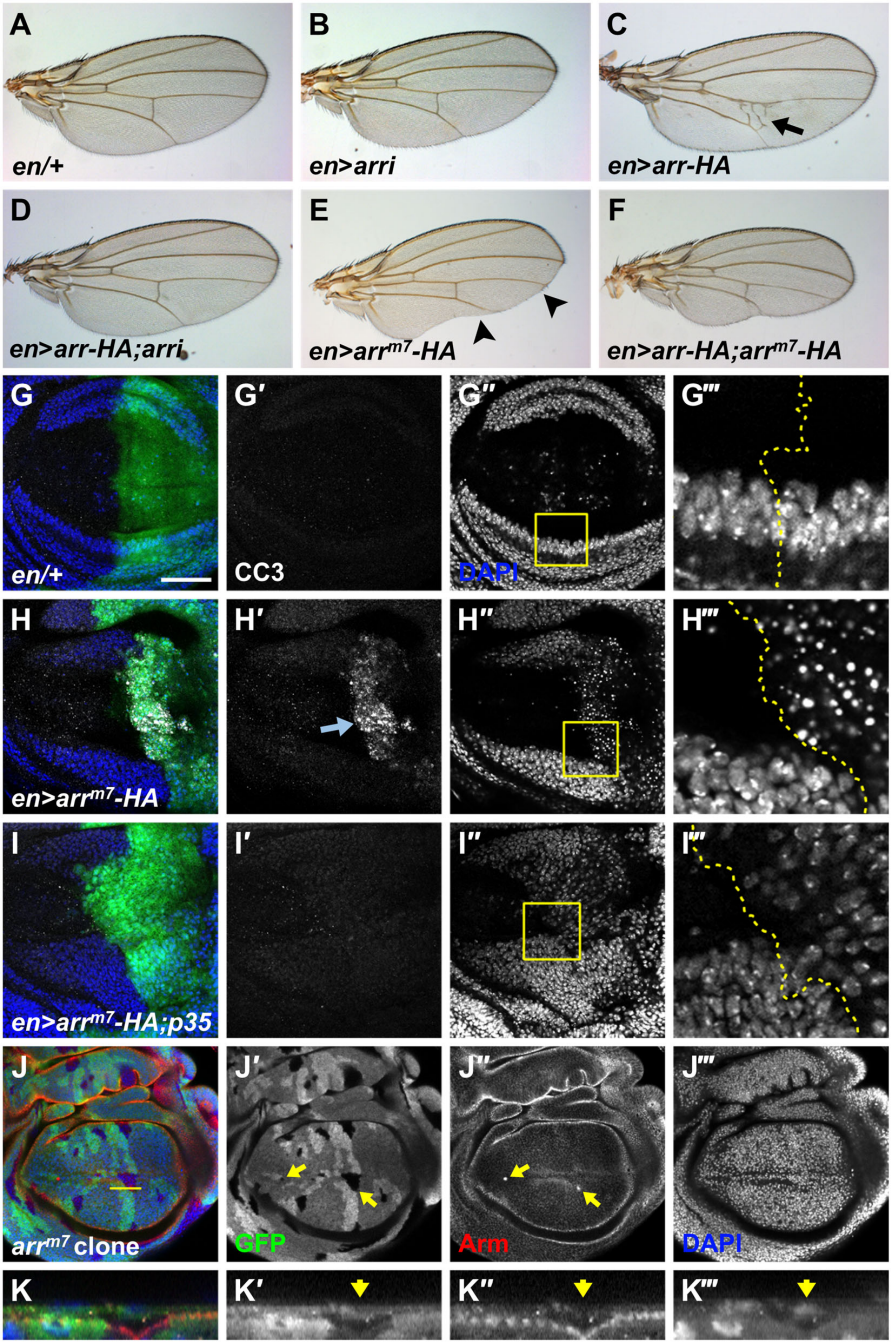
*arr*^*m7*^ is dominant negative and induces cell death. **a**–**f** *en-Gal4; UAS-GFP* flies were crossed with *UAS* lines and wings of their progenies are shown (*n* = 20, each). Genotypes are written at the lower left without GFP. Extra cross-veins are marked with an arrow in (**c**). Notched wing is marked with arrowheads in (**e**). These wing phenotypes showed 100% penetrance. **g**–**i** *UAS* lines were crossed with *en-Gal4; UAS-GFP* flies and wing discs of their progenies were stained. Images were taken by focusing at the basal position. The *en* expressing posterior regions are indicated with GFP. The regions marked with yellow squares are magnified and the borders of GFP regions are indicated with dotted lines in (**g‴**,**h‴**,**i‴**). CC3 is marked with an arrow in (**h′**). **j**, **k** Clonal analysis of *arr*^*m7*^ generated at the 2nd larvae stage by heat shock for 45–50 min at 37 °C. Twin spots (+/+) have the highest level of GFP, heterozygous cells (+/m) have the intermediate level of GFP, and *arr*^*m7*^ clones (m/m) have no GFP. Accumulated Arm in *arr*^*m7*^ clones are indicated by arrows. One cross-section at the region marked with a yellow line is shown in (**k**). Only the apical region is shown. Scale bar, 50 μm.

We utilized *UAS-arr-HA* and *UAS-arr*^*m7*^*-HA* flies to compare Arr and Arr^m7^ proteins in western analysis with the anti-Arr antibody^32^. The epitope of the anti-Arr antibody was present in the intracellular region of Arr that is missing in Arr^m7^-HA (Fig. S2A, B). Accordingly, Arr-HA protein was recognized by both anti-HA and anti-Arr antibodies in *engrailed* (*en*)*>arr-HA* wing discs whereas Arr^m7^-HA protein was recognized by only anti-HA antibody in *en* > *arr*^*m7*^*-HA* wing discs (Fig. S2C–F). We also found that *en* > *arr RNAi* wings are normal despite substantial reduction in the amount of Arr, indicating that *arr RNAi* expression still leaves behind a low level of Arr enough for normal development (Fig. 2a, b and S2G). Similar phenomenon with the same *UAS-arr RNAi* flies has been reported elsewhere^33^.

Extra cross-veins were generated in all *en* > *arr-HA* wings examined, and fully rescued in *en* > *arr-HA; arr RNAi* wings (Fig. 2a–d). Unlike *en* > *arr-HA* wings, notched wings were generated in all *en* > *arr*^*m7*^*-HA* wings examined (Fig. 2e). Arr^m7^ expression by *nubbin-Gal4* also induced wing notching, and that by *patched-Gal4* resulted in pupal lethality (Fig. S3). When *arr-HA* and *arr*^*m7*^*-HA* were co-expressed by *en-Gal4*, both extra cross-veins and wing notching were fully rescued (Fig. 2f). Similarly, pupal lethality of *ptc* > *arr*^*m7*^*-HA* flies was rescued by co-expression with *arr-HA* (data not shown). Therefore, phenotypes induced by Arr-HA was suppressed by co-expression with Arr^m7^-HA and vice versa. This suggests that dimerization between Arr and Arr^m7^ leads to decreased Arr activity due to dominant-negative Arr^m7^. Our result is consistent with *Xenopus* LRP6ΔC equivalent to Arr^m7^, which shows dominant negativity in axis formation^34^.

### arr^m7^ induces cell death

Wing notching induced by *arr*^*m7*^*-HA* overexpression may be due to cell death because Sona is important for cell survival^19^. Indeed, cleaved caspase 3 (CC3) and basally positioned pyknotic nuclei due to chromatin condensation were detected in *en* > *arr*^*m7*^*-HA* but not in *en-Gal4/+* wing discs^35^ (Fig. 2g, h). *en* > *arr*^*m7*^*-HA*; *p35* wing discs had neither CC3 nor pyknotic nuclei, although normal-looking nuclei were still present at the basal position (Fig. 2i). Therefore, the caspase inhibitor p35 partially rescued cell death phenotype induced by *arr*^*m7*^*-HA* expression.

We also carried out clonal analysis of *arr*^*m7*^ allele. When formation of *arr*^*m7*^ clones was induced during first instar larval stage, no clones but only twin spots were detected in the wing pouch region (Fig. S4). *arr*^*m7*^ clones were detected when induced during second instar larval stage (Fig. 2j). The twin spots were larger than the clones, which is consistent with cell death phenotype of the *arr*^*m7*^ allele. Some *arr*^*m7*^ clones had a spot with higher level of Arm at one edge because *arr*^*m7*^ cells slightly invaginated (Fig. 2k). Similar but severer phenotype was observed with *arr*^*2*^ clones, in which *arr*^*2*^ cells completely invaginate and extruded from the basal side^36^. Thus, we concluded that the loss of *arr* induces cell death.

### arr clones exhibit higher level of extracellular Wg whereas lower level of extracellular Sona

We tested further whether *arr*^*m7*^ clones show phenotypes similar to *arr*^*2*^ clones. As shown with *arr*^*2*^ clones^37,38^, the level of extracellular Wg was increased in *arr*^*2*^ MARCM clones (Fig. S5A). We also found that the level of intracellular Wg is slightly increased in *arr*^*2*^ MARCM clones (Fig. S5B). Similarly, *arr*^*m7*^ clones had higher levels of both extra- and intra-cellular Wg than surrounding cells (Fig. S5C, D).

**Fig. 3:**
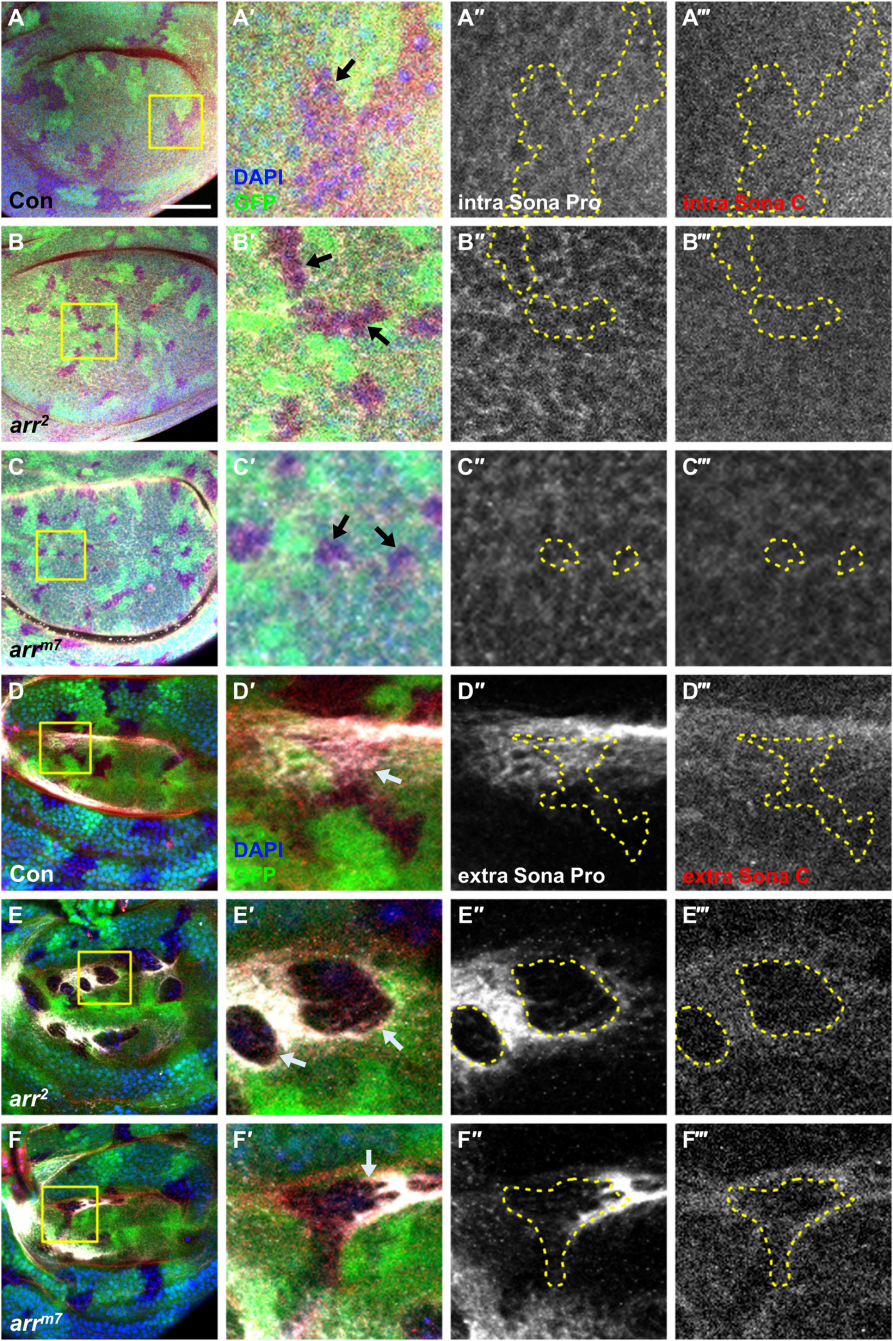
*arr*^*2*^ and *arr*^*m7*^ clones show increase in the level of extracellular Sona. Clones were generated at the 2nd larvae stage by heat shock at 37 °C for 45–50 min. Sona was visualized with two antibodies, Sona-Pro and Sona-C. Regions marked with the yellow squares are magnified. Clone boundaries are marked by yellow dots and clones are marked with arrows. Images were taken at the apical position for intracellular Sona as shown by DAPI. **a**–**c** Control, *arr*^*2*^ and *arr*^*m7*^ clones with no change in intracellular level of Sona. **d** A control clone with no change in extracellular level of Sona. **e** *arr*^*2*^ clones with lower level of extracellular Sona. **f** *arr*^*m7*^ clones with lower level of extracellular Sona. Scale bar, 50 μm.

Given the genetic interaction between *arr* and *sona*, we reasoned that Arr may affect the level of intracellular or extracellular Sona. *arr*^*2*^ and *arr*^*m7*^ clones as well as control clones showed no obvious change in the level of intracellular Sona detected by Sona-Pro and Sona-C antibodies that recognize Sona pro domain and carboxyl region, respectively (Fig. 3a–c). When control clones were formed in the blade region where the level of extracellular Sona is high, no change was detected in the extracellular level of Sona (Fig. 3d). It was clear, however, that the levels of extracellular Sona in *arr*^*2*^ and *arr*^*m7*^ clones were lower than neighboring wild-type cells (Fig. 3e, f). Therefore, Arr is important for maintaining the level of extracellular Sona in vivo.

### Arr increases both intracellular and extracellular levels of Sona in S2 cells

To understand the mechanism by which Arr regulates the level of extracellular Sona in vivo, we utilized S2 cell culture system. S2 cells co-transfected with *UAS-sona*, *UAS-arr* and *act-Gal4* plasmids were cultured, and then S2 cells were used to prepare cell extract (CX), and conditioned media were used to prepare two fractions, P100 and SN_Δ_ fractions. The P100 fraction containing EVs was obtained as a pellet after centrifuging precleared media at 100,000×*g*, and the SN_Δ_ fraction containing soluble secreted proteins was obtained as a supernatant^29^. As previously shown, 70 kDa full-length Sona is processed to a 37 kDa active form of Sona in both intra- and extracellular regions, and 22 kDa fragments processed from the pro domain is co-secreted with the 37 kDa form of active Sona^17^.

**Fig. 4:**
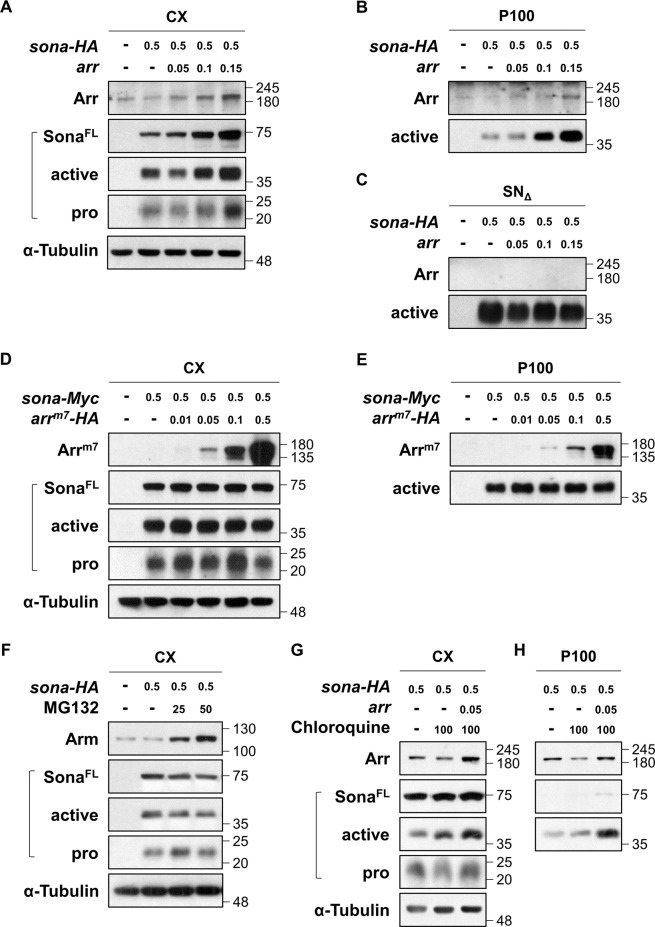
Arr but not Arr^m7^ increases the level of Sona. Anti-Arr, Sona-Pro, anti-HA, and anti-α-Tubulin antibodies were used as blotting antibodies to visualize Arr, full-length Sona (Sona^FL^), active Sona (active), 22 kDa pro domain fragment (pro) and α-Tubulin, respectively. S2 cells were co-transfected with *sona* and *arr* cDNAs with *actin-Gal4* and cultured for 3 days before sampling. Amounts of transfected DNA in μg are indicated. **a**–**c** S2 cells were transfected with a constant amount of *sona-HA* DNA and increasing amounts of *arr* DNA. CX, P100, and SN_Δ_ fractions are analyzed in (**a**–**c**), respectively. **d**, **e** Transfection of a constant amount of *sona-Myc* and increasing amounts of *arr*^*m7*^*-HA*. CX and P100 fractions are analyzed. **f**–**h** Effects of MG132 (μM) in (**f**), and chloroquine (μM) in (**g**, **h**) on the amount of Sona. The α-Tubulin is a loading control for CX.

S2 cells do not express Sona or Wg^39,40^ whereas expressed Arr (Fig. 4a). When S2 cells were co-transfected with a constant amount of *UAS-sona* plasmid and varying amounts of *UAS-arr* plasmid together with *act-Gal4* plasmid, amounts of 70, 37, and 22 kDa Sona forms were all increased in correlation with the amount of Arr in CX (Fig. 4a). Such increase was not due to increase in the transcription rate of *sona* by Arr because transcription of transfected *sona* cDNA in S2 cells is under the control of Gal4 driven by the *actin* promoter. This established that Arr increases the stability of Sona.

We then examined whether Arr increases the level of extracellular Sona in P100 and SN_Δ_ fractions. In fact, the level of active Sona in the P100 fraction was increased whereas that in the SN_Δ_ fraction was not changed with increasing amounts of Arr (Fig. 4b, c). Therefore, Arr specifically increased the level of Sona in P100. One interesting finding was that Arr itself is present in the P100 fraction but not in the SN_Δ_ fraction, which will be addressed later (Fig. 4b, c). We also checked whether Arr^m7^ changes the level of Sona. The level of all Sona forms in CX and P100 were unchanged when increasing amounts of Arr^m7^ were co-expressed in S2 cells (Fig. 4d, e). Therefore, the intracellular domain of Arr is essential for stabilization of Sona.

We then asked whether Wg has any effects on this new function of Arr (Fig. S6). When constant amounts of *GFP-wg* and *sona* plasmids and increasing amounts of *arr* plasmid were co-expressed, the levels of all Sona forms were unchanged regardless of Arr. Therefore, Wg somehow eliminated the effect of Arr on the level of Sona so we concluded that Wg is not involved in the stabilization of Sona by Arr.

### Arr increases the level of Sona by inhibiting lysosomal degradation pathway

Proteins are degraded by two pathways, proteasomal degradation pathway and lysosomal degradation pathway^41,42^. To examine which pathway is regulated by Arr for stabilizing Sona, MG132 and chloroquine were used to inhibit proteasomal degradation and lysosomal pathways, respectively^43^. MG132 was added to culture media to final concentrations of 25 and 50 μM for 6 h before sampling, and the amount of Arm as a positive control was increased in correlation with the amount of MG132^44,45^ (Fig. 4f). In contrast, MG132 did not change levels of any Sona forms. This showed that proteasomal degradation pathway does not affect stability of Sona.

Treatment of Sona-expressing S2 cells with 100 μM chloroquine for 24 h prior to sampling did not noticeably change the amount of Sona in CX compared to untreated Sona-expressing S2 cells (Fig. 4g). When Sona and Arr were co-expressed in chloroquine-treated S2 cells, however, amounts of Sona forms in CX were slightly increased, and the amount of active Sona in P100 was prominently increased (Fig. 4h). Chloroquine treatment even made the full-length Sona detectable although full-length Sona is usually undetectable in P100^20^ (Fig. 4h). Therefore, we concluded that Arr increases stability of Sona by inhibiting the lysosomal degradation pathway.

### Sona neither cleaves Arr nor changes the level of Arr

It has been proposed that the extracellular region of Arr is processed by an unknown metalloprotease based on the detection of intracellular fragments of Arr^46^. To test whether Sona processes Arr, the same amount of *arr-HA* with increasing amounts of *sona-Myc* were expressed in S2 cells. The amount of full length Arr in CX or P100 was not affected by Sona, indicating that Sona cleaves neither intracellular nor extracellular Arr (Fig. S7A, B). We also tested whether Sona affects the level of Arr in vivo. When Sona was transiently expressed in *dpp* > *sona* wing discs for only 24 h using Gal80^ts^ in order to avoid lethality by Sona overexpression, the level of neither Arr-LacZ nor Arr protein changed (Fig. S7C, D). In sum, Sona does not affect transcription, translation and post-translation of *arr*.

### Arr and Sona are present in the same protein complex and co-localize in wing disc cells

**Fig. 5:**
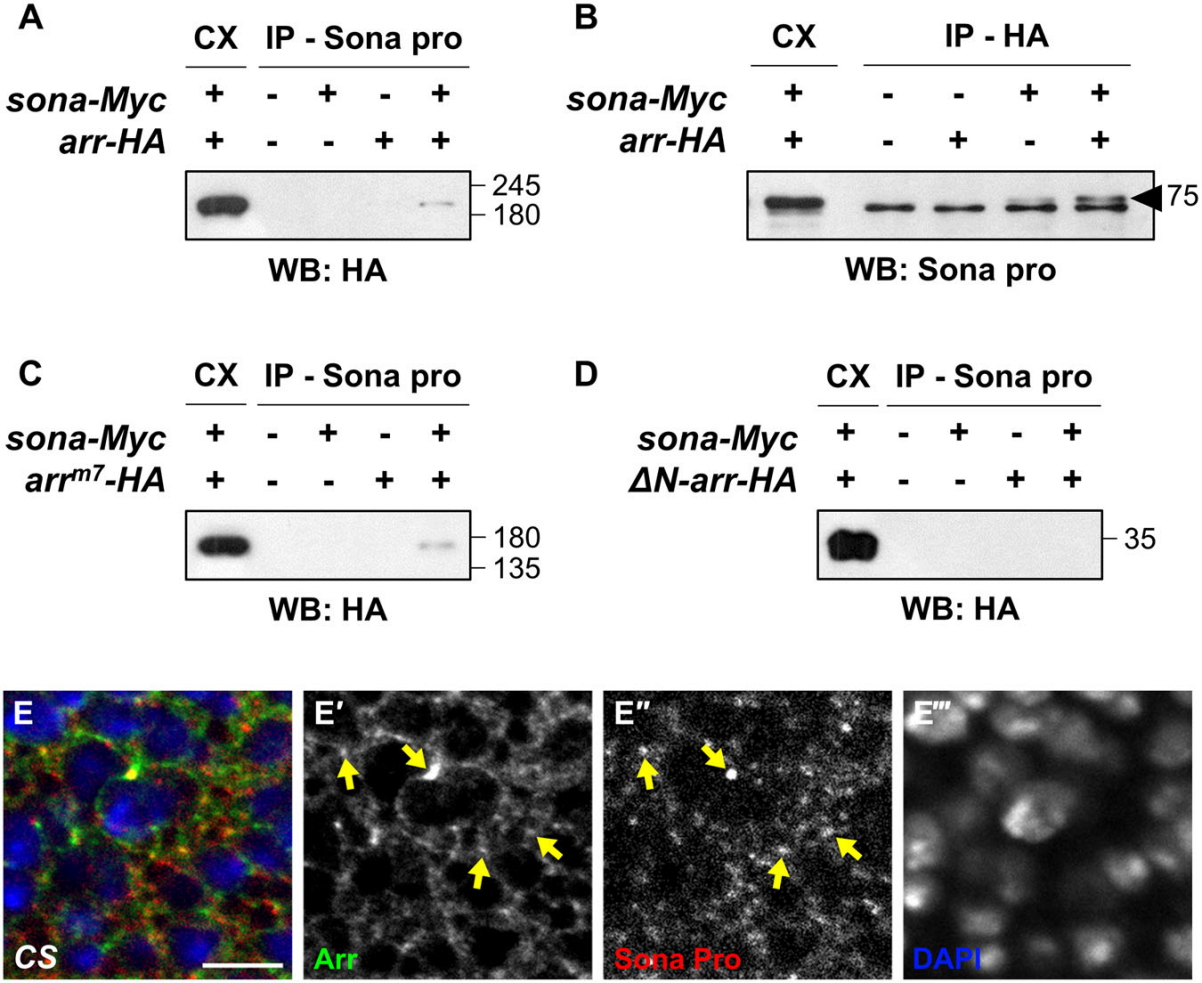
Arr and Sona are present in a same protein complex and co-localize in wing disc cells. **a**, **b** Sona-Myc and Arr-HA are co-IPed. Arr-HA was detected when Sona-Myc was IPed in **a**, and vice versa in (**b**). The arrowhead indicates full length Sona above non-specific bands. **c**, **d** Co-IP of Sona with Arr-HA requires the extracellular domain of Arr. Arr^m7^-HA in (**c**), but not ΔN-Arr-HA in (**d**) is co-IPed with Sona-Myc. **e** A wing disc of *CS* was stained for Arr and Sona and an image of a region near the DV midline was taken. Arrows mark a few vesicular structures with both Arr and Sona. Scale bar, 5 μm.

The biochemical relationship between Arr and Sona prompted us to examine whether they are present in the same protein complex. We co-expressed *sona-Myc* and *arr-HA* in S2 cells, and the CX of these S2 cells was used for co-immunoprecipitation (co-IP) analysis. Arr-HA was detected after precipitation with Sona-Pro antibody, and full-length Sona was detected after precipitation with anti-HA antibody, indicating that intracellular Sona and Arr are present in the same protein complex (Fig. 5a, b). To find which domain of Arr is responsible for co-IP with Sona, Arr^m7^-HA or ΔN-Arr-HA was co-expressed with Sona in S2 cells (Fig. 1). Arr^m7^-HA was co-IPed but ΔN-Arr-HA was not with Sona-Myc, showing that the extracellular domain of Arr is involved in interaction with Sona (Fig. 5c, d). We also found that Arr and Sona co-localize in some intracellular vesicular structures (Fig. 5e).

### Arr is present in the exosome fraction

**Fig. 6:**
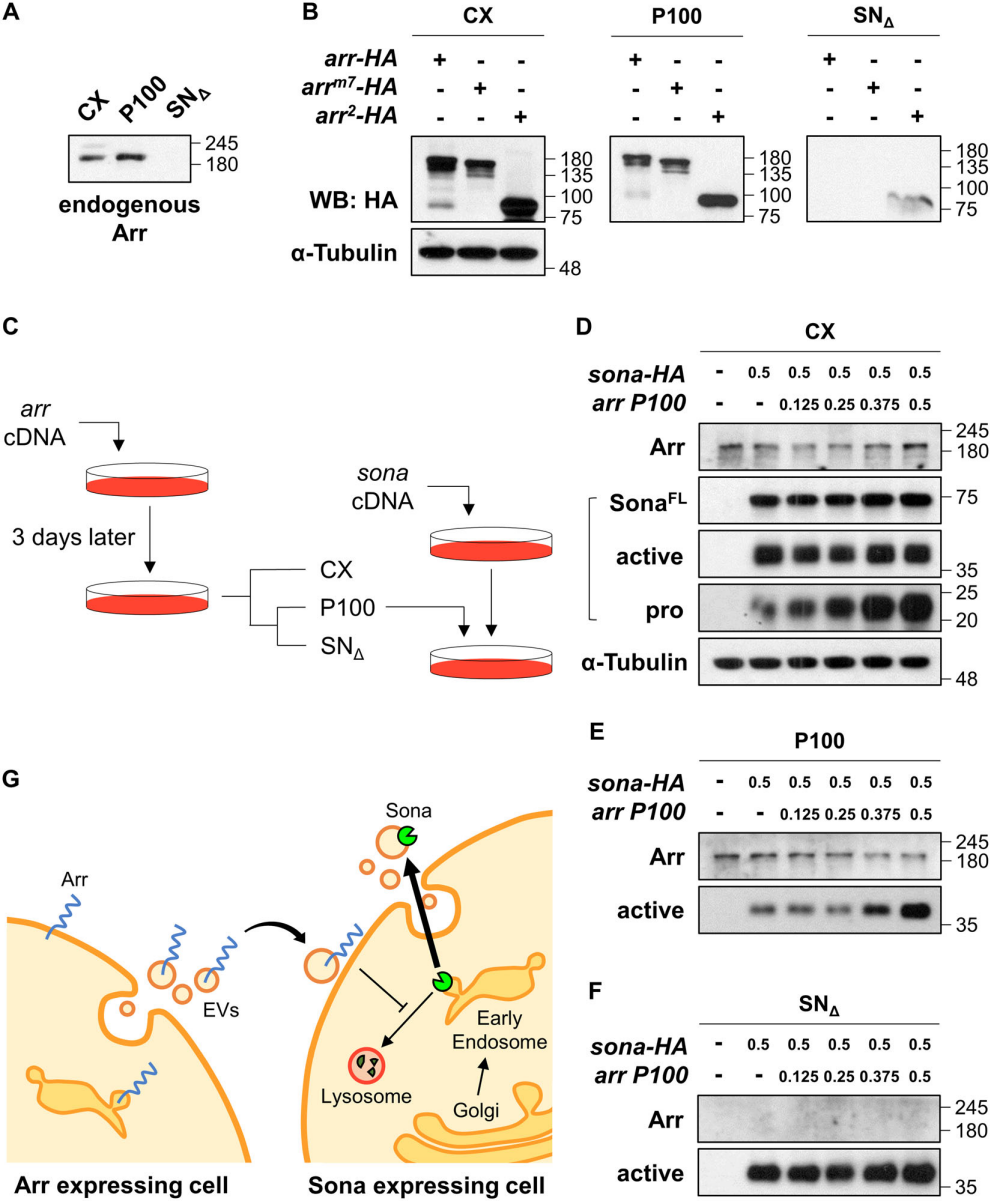
Arr secreted via EVs increases the level of exosomal Sona. **a** Endogenous Arr expressed in S2 cells are secreted via EVs. Arr is present in the P100 but not in the SN_Δ_ fraction. **b** Arr-HA, Arr^m7^-HA, and Arr^2^-HA are secreted via EVs. S2 cells were transfected with cDNAs encoding different Arr forms and their CX, P100, and SN_Δ_ fractions were analyzed. **c** A scheme of the experiments in (**d**–**f**). S2 cells were transfected with different amounts of *arr* cDNAs, cultured for 3 days, and then were used to prepare the P100 fraction. Varied amounts of this P100 fraction was added to S2 cells that had been transfected with *sona* cDNA and cultured for 3 days. After 3 days of culture, S2 cell culture was used to prepare CX, P100, and SN_Δ_ fractions. **d**–**f** Amounts of transfected *sona-HA* and *arr* DNAs were indicated. The α-Tubulin is a loading control for CX. **g** A model to explain a new function of Arr for regulating the level of extracellular Sona. Arr is secreted via EVs, and this exosomal Arr acts to send signal to early endosomes to make Sona to take secretion via EVs or channeled to lysosomal degradation pathway.

We detected Arr in the P100 fraction prepared from S2 cells transfected with *arr* cDNA (Fig. 4b). Endogenous Arr in S2 cells was also present in the P100 fraction but not in the SN_Δ_ fraction, demonstrating that Arr is secreted to extracellular space as tethered to EVs (Fig. 6a). To examine which region of Arr is essential for dictating Arr to EVs, S2 R+ cells transfected with *arr*, *arr*^*m7*^, or *arr*^*2*^ cDNAs were cultured for 3 days, and CX, P100, and SN_Δ_ fractions were prepared. Like endogenous Arr, overexpressed Arr-HA and Arr^m7^ were detected in the P100 fraction but not in the SN_Δ_ fraction (Fig. 6b). Arr^2^ protein retaining only the amino terminal half was also detected in P100 fraction with a slight amount in SN_Δ_ fraction (Fig. 1a). Taken together, the extracellular region of Arr is sufficient for secretion of Arr.

### Exosomal Arr increases the extracellular level of Sona

To examine whether exosomal Arr affects the levels of intracellular and extracellular Sona, we carried out an experiment (Fig. 6c). S2 cells were transfected with *arr* cDNA and cultured for 3 days to obtain conditioned media, from which the P100 fraction was prepared. This P100 fraction was then added to fresh culture media of S2 cells immediately after *sona* transfection. After 3 days culture, these cells and conditioned media were used to prepare CX, P100 and SN_Δ_ fractions for comparing the amounts of Sona in these fractions. As a negative control, the P100 fraction prepared from S2 cells transfected with the vector were used.

The addition of the Arr-containing P100 fraction to Sona-expressing cell culture noticeably increased the level of the 22 kDa fragment in CX whereas the amount of other Sona forms was not changed (Fig. 6d). On the other hand, the amount of active Sona in the P100 fraction increased in correlation with that of Arr (Fig. 6e). Such increase was not detected in the SN_Δ_ fraction (Fig. 6f). Thus, exosomal Arr somehow stabilizes Sona in cytoplasm by inhibiting lysosomal pathway, which eventually increases the level of active Sona in EVs.

## Discussion

We report here that the loss of *arr* decreases the level of extracellular Sona in flies, and Arr increases the extracellular level of Sona by stabilizing intracellular Sona in S2 cells through inhibition of lysosomal pathway. Interestingly, Arr was present in the fraction containing exosomes, and the exosomal Arr also increases the level of extracellular Sona by unknown mechanisms. We propose that exosomal Arr inhibits lysosomal degradation pathway and increases the level of exosomal Sona. When Arr is absent, Sona enters the lysosomal pathway to be degraded (Fig. 6g).

The *m7* suppressor with both *arr*^*m7*^ and *pdm3*^*m7*^ was identified in the screen using ethyl methanesulfonate (EMS) as a mutagen^17^. It is worth noting that *arr*^*m7*^ has a G to A transition, whereas *pdm3*^*m7*^ has an insertion of a defective *hobo* element in the untranslated region of the *pdm3* gene^16^ (Fig. 1a). The point mutation in the *arr* gene seemed to occur before the insertion of the *hobo* element in the *pdm3* gene, because other suppressors did not have *hobo* elements in their *pdm3* genes. Therefore, both inhibition of *wg* transcription by *pdm3*^*m7*^ and decrease in the level of extracellular Sona by *arr*^*m7*^ may have been crucial for the *m7* suppressor to be identified in the screen.

Arr^m7^ protein does not have the intracellular domain but still has the LDLR domain involved in dimerization, so dimers formed between Arr^m7^ and wild-type Arr in *arr*^*m7*^ heterozygotes become inactive. Besides this Arr-Arr^m7^ dimer, Arr-Arr and Arr^m7^-Arr^m7^ dimers are also formed in *arr*^*m7*^ heterozygotes, and the amount of Arr-Arr dimers may be enough to support normal development of *arr*^*m7*^ heterozygotes. This explanation is in line with the finding that *en* > *arr RNAi* flies develop normally despite the low level of Arr protein in these flies (Fig. 2b). Although *arr RNAi* expression induced no visible phenotypes in our experiments, it suppressed phenotypes induced by overexpression of Arr or Sona (Figs. 1b and 2d).

All biochemical experiments in this report were carried out with S2 cells that do not express Wg^39,40^, indicating that stabilization of Sona by Arr occurs in the absence of Wg (Fig. 4). Similar conclusion was drawn in case of LRP6 and gap junction protein connexin 43 (Cx43) in cardiac gap junction assembly^47^. Transcription of *Cx43* gene is induced by Wnt signaling independent of LRP6, but instead LRP6 post-transcriptionally promotes traffic of Cx43 from endoplasmic reticulum to Golgi apparatus. Reduction of LRP6 leads to retention of Cx43 in ER, which leads to the lysosomal degradation of immature Cx43. Such relationship between LRP6 and Cx43 is remarkably similar to that of Arr and Sona. It has been also reported that LRP6 deficiency results in lethal dilated cardiomyopathy and cardiac dysfunction by activation of dynamin-related protein 1 signaling^48^. Therefore, Arr/LRP6 seems to play new post-transcriptional roles independent of Wnt signaling.

We have shown here that coexpression of Arr and Sona in S2 cells stabilizes Sona and increases the level of exosomal Sona by inhibiting the lysosomal degradation pathway. In addition, Arr is present in exosomes and these exosomal Arr also stabilizes Sona. It is actually common to find Wg signaling components in EV populations such as Wnts, β-catenin/Arm, Wls, and Fzd-10^49^. LRP6 is also found in the exosome fraction by proteome profiling, although further confirmation is required^50,51^. It seems that both Arr/LRP6 and Fz are secreted via EVs, and interaction between the Wnt co-receptors and Wnt in EVs may promote Wnt signaling but may also perform extracellular EV-specific functions independent of Wnt signaling as reported here. Further understanding mechanisms of secreted exosomal Arr in stabilization of Sona will greatly help to reveal Wnt-independent functions of Arr.

## Supplementary information

Figure S1

Figure S2

Figure S3

Figure S4

Figure S5

Figure S6

Figure S7

Supplementary Figure legends
