# Supplementary figures and images for: Exosomal arrow (Arr)/lipoprotein receptor protein 6 (LRP6) in Drosophila melanogaster increases the extracellular level of Sol narae (Sona) in a Wnt-independent manner
Source: Cell Death Dis. 2020 Nov 3;11(11):944. doi: 10.1038/s41419-020-02850-x (PMC7608652; doi:10.1038/s41419-020-02850-x)

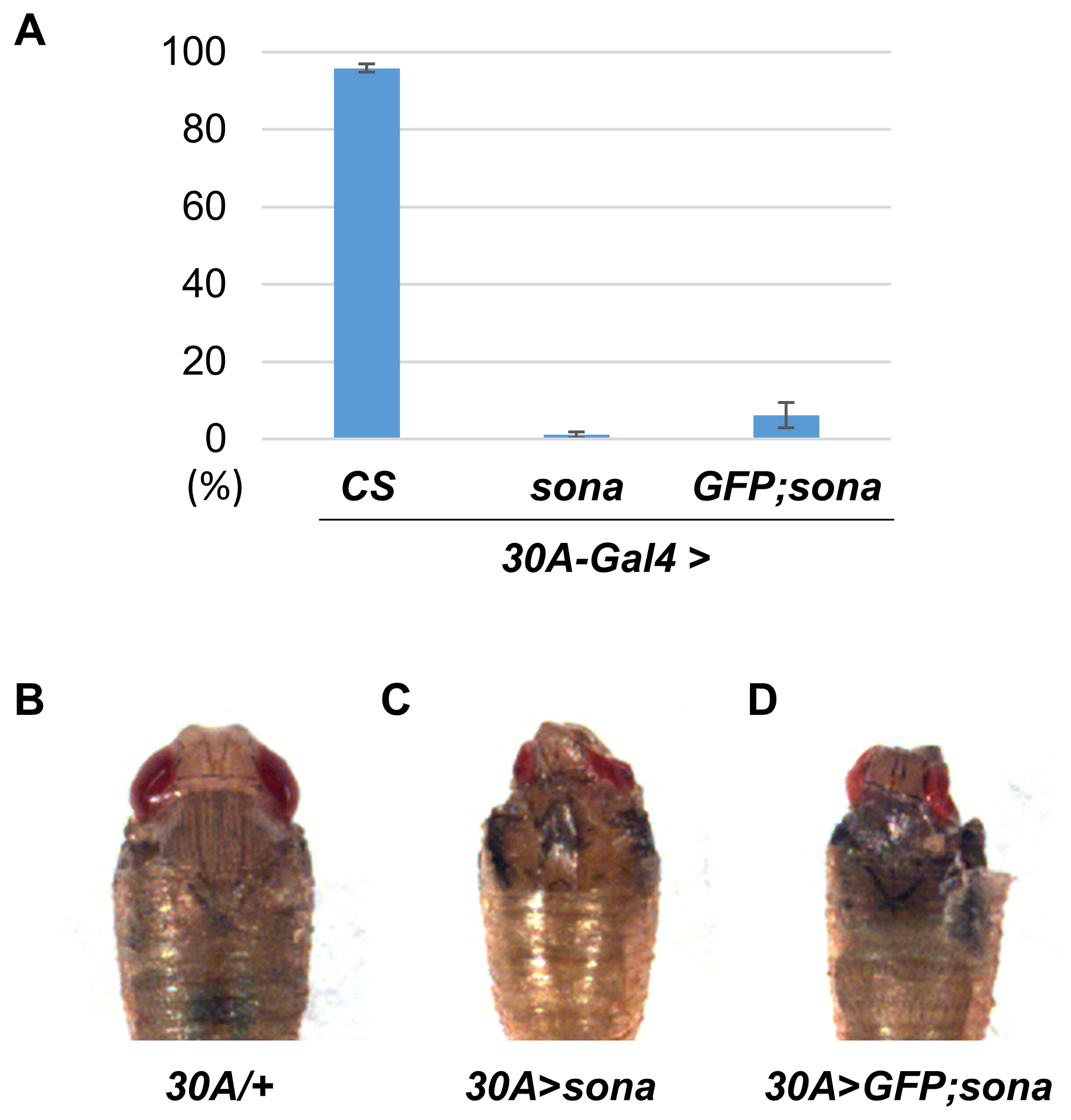

Supplement: Supplementary file 1 — Figure S1 [file 41419_2020_2850_MOESM1_ESM.png]

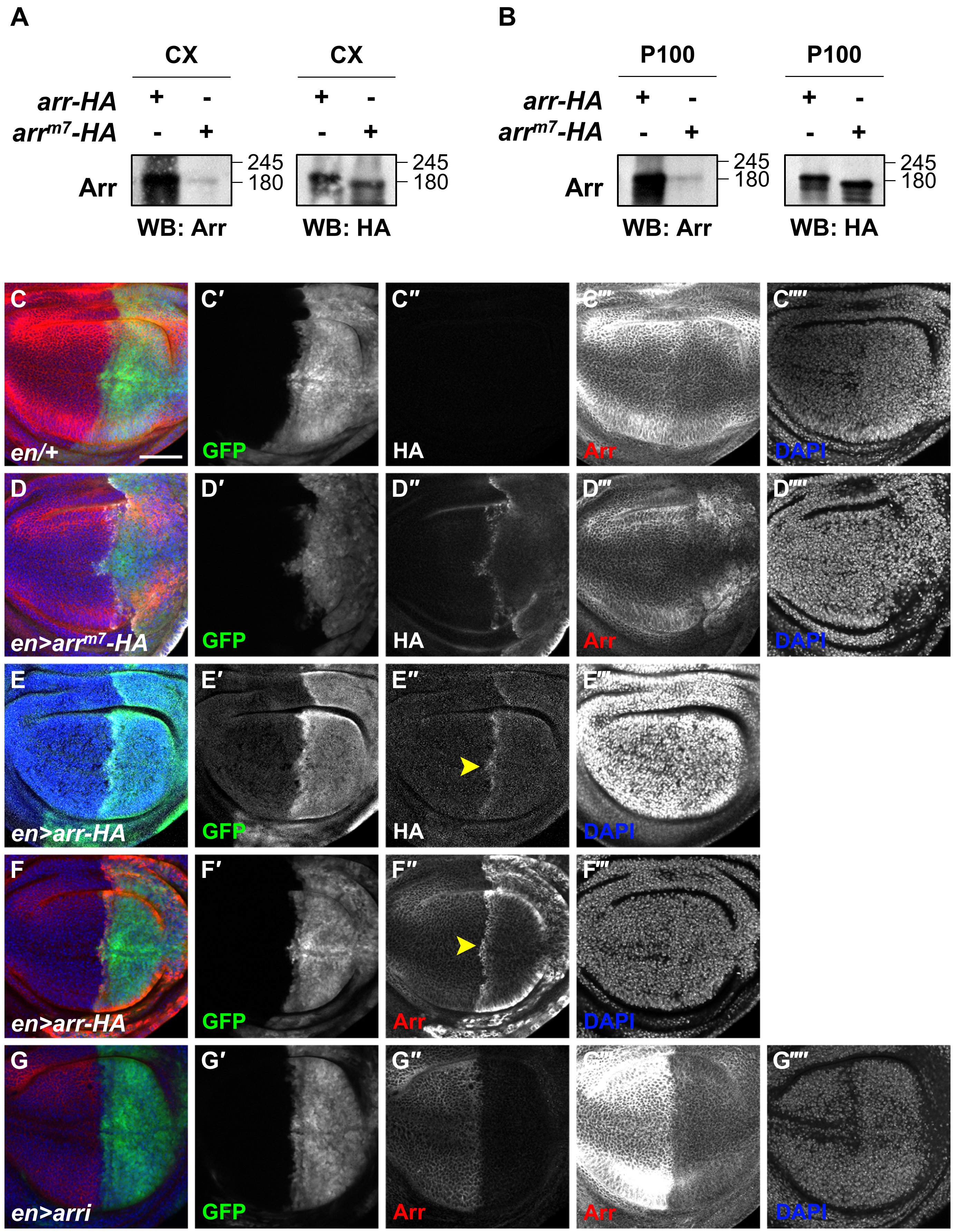

Supplement: Supplementary file 2 — Figure S2 [file 41419_2020_2850_MOESM2_ESM.png]

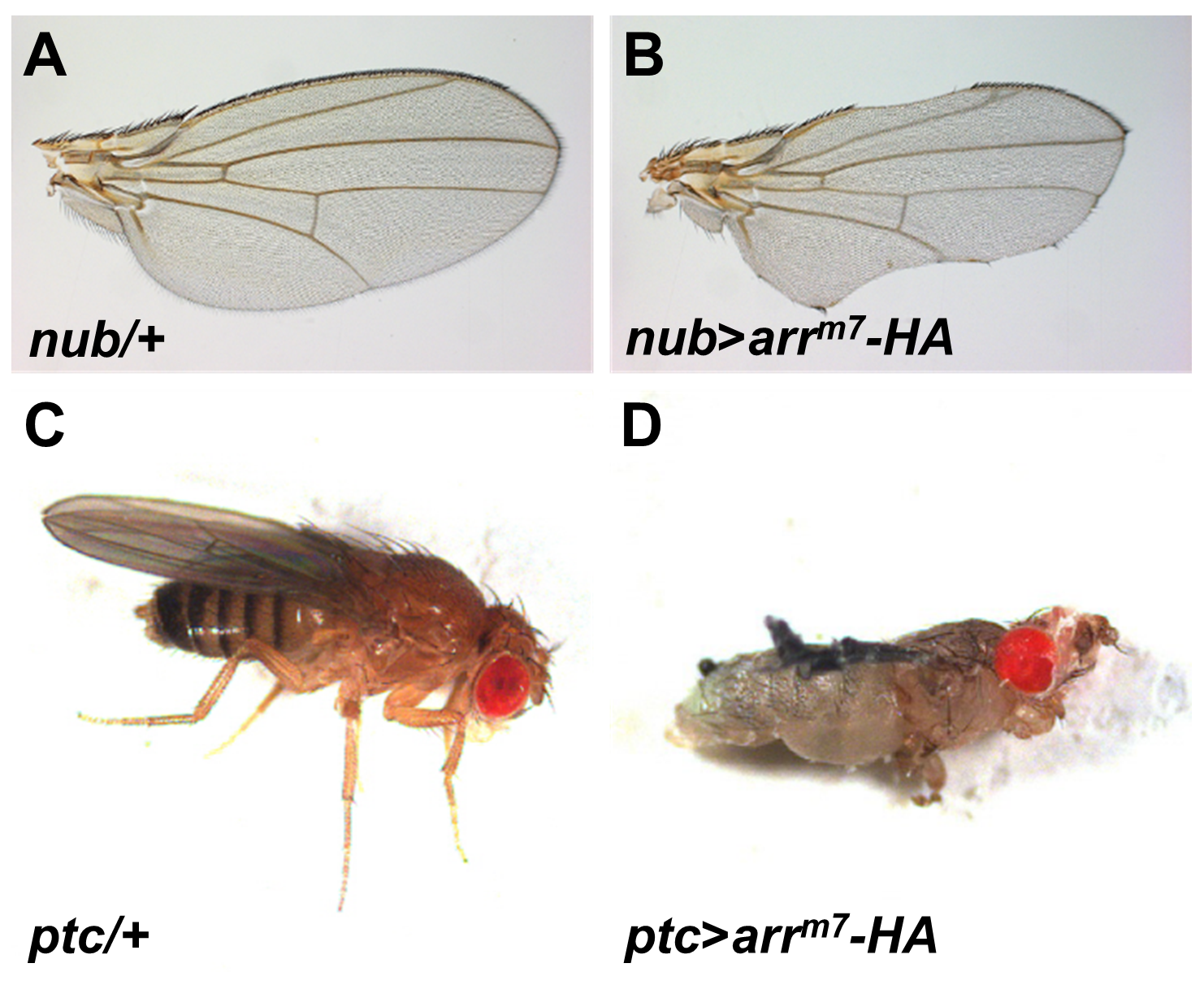

Supplement: Supplementary file 3 — Figure S3 [file 41419_2020_2850_MOESM3_ESM.png]

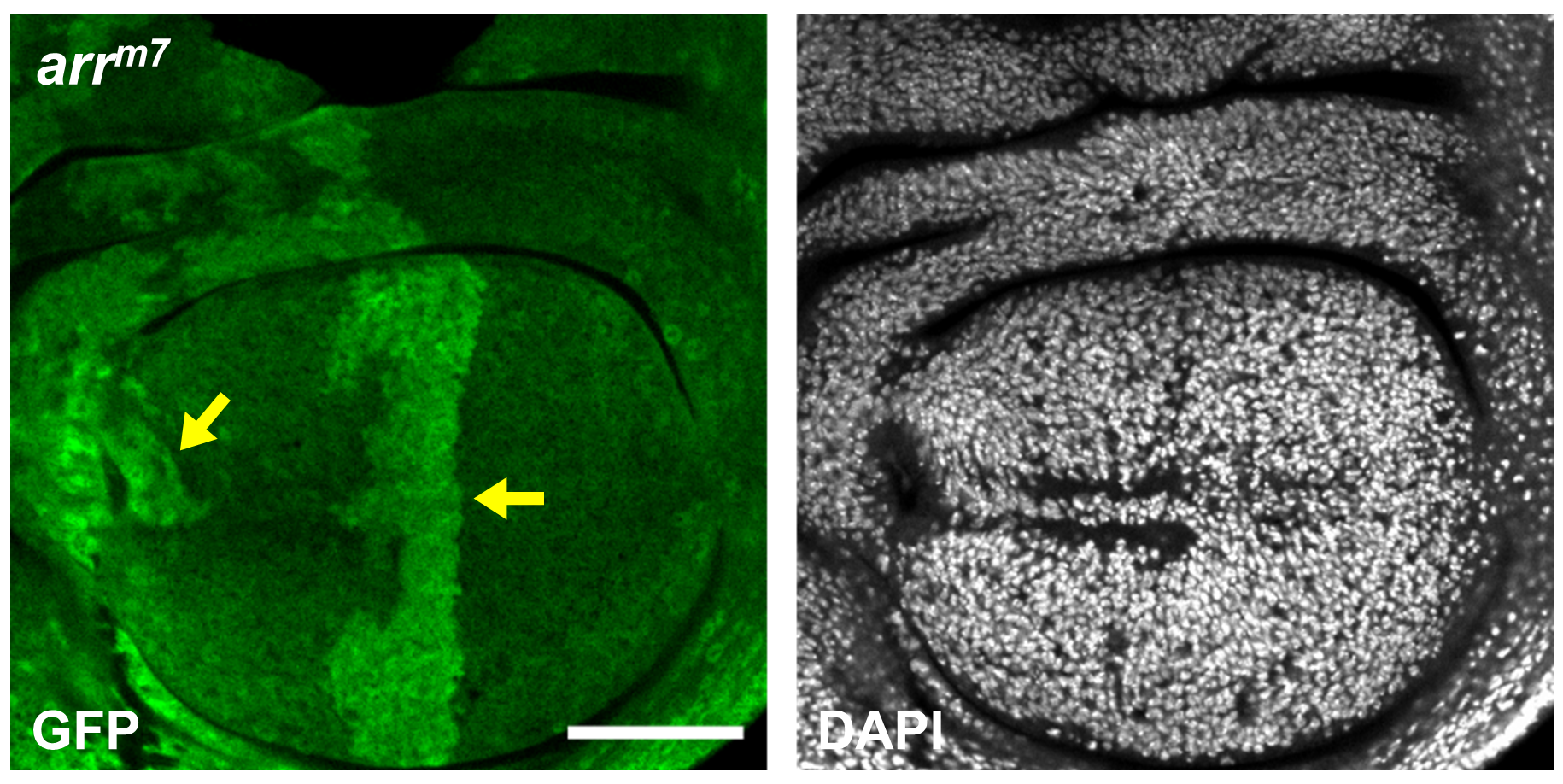

Supplement: Supplementary file 4 — Figure S4 [file 41419_2020_2850_MOESM4_ESM.png]

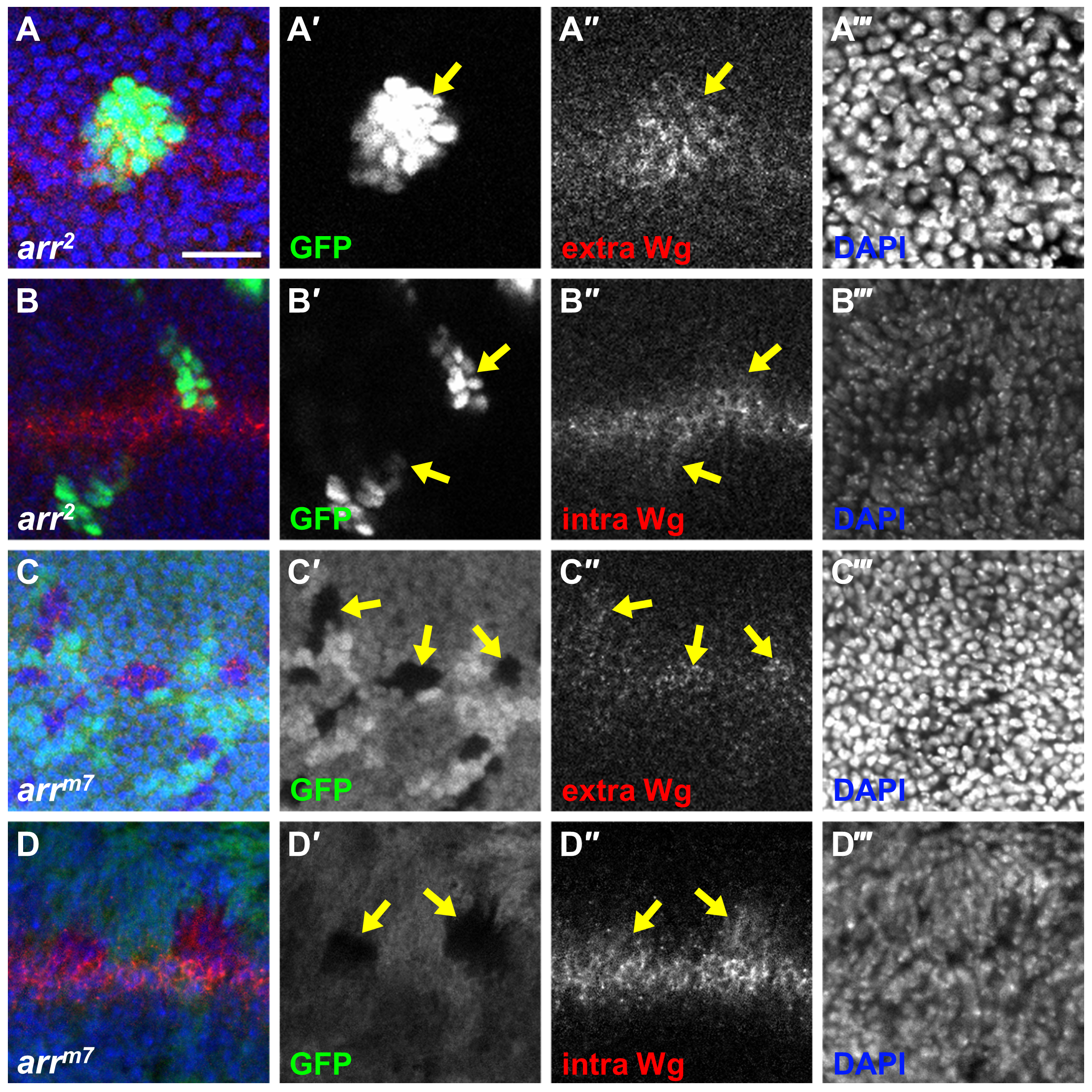

Supplement: Supplementary file 5 — Figure S5 [file 41419_2020_2850_MOESM5_ESM.png]

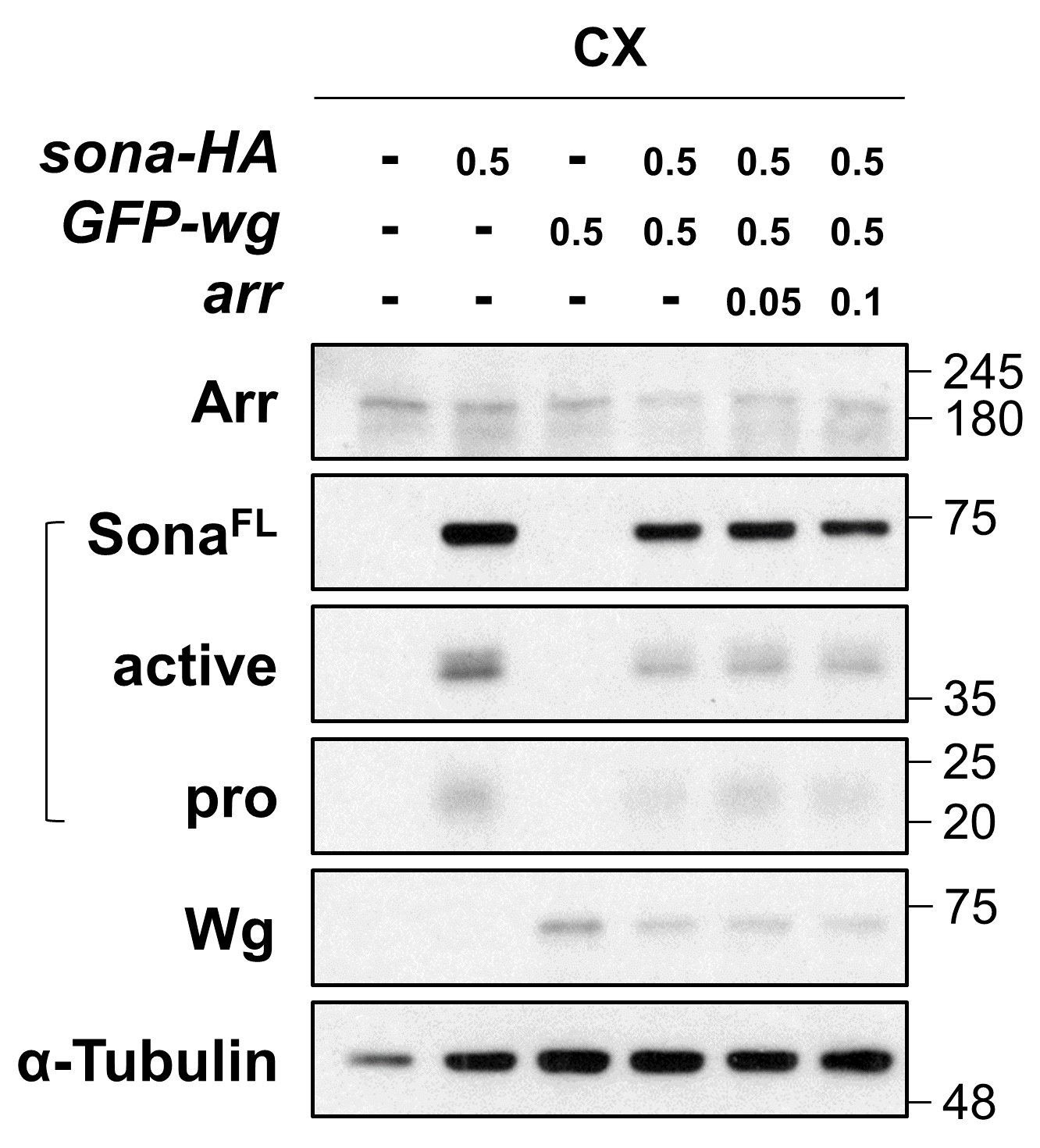

Supplement: Supplementary file 6 — Figure S6 [file 41419_2020_2850_MOESM6_ESM.png]

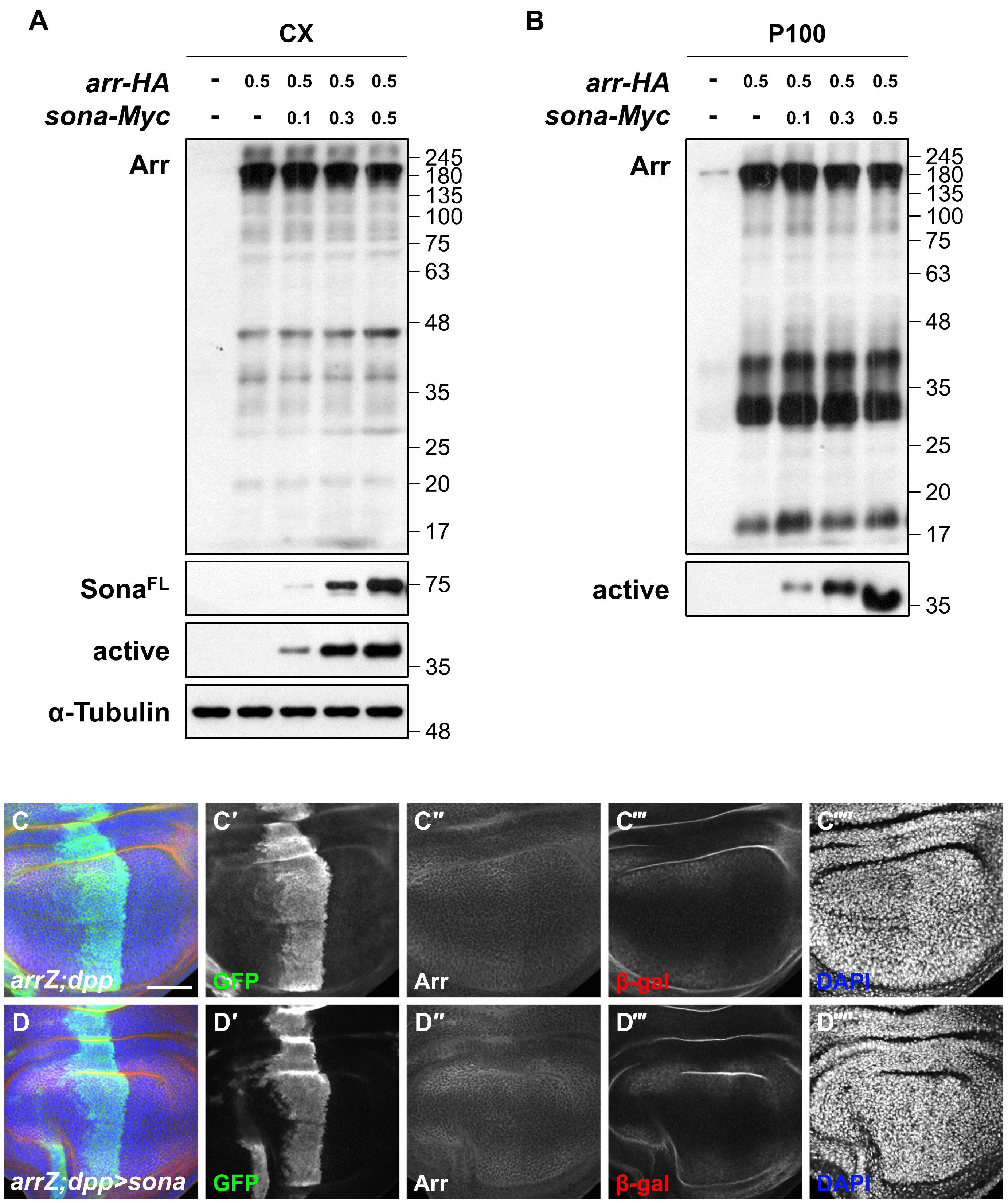

Supplement: Supplementary file 7 — Figure S7 [file 41419_2020_2850_MOESM7_ESM.png]
