# Supplementary material for: Exosomal arrow (Arr)/lipoprotein receptor protein 6 (LRP6) in Drosophila melanogaster increases the extracellular level of Sol narae (Sona) in a Wnt-independent manner
Source: Cell Death Dis. 2020 Nov 3;11(11):944. doi: 10.1038/s41419-020-02850-x (PMC7608652; doi:10.1038/s41419-020-02850-x)
Supplement: Supplementary file 8 — Supplementary Figure legends [file 41419_2020_2850_MOESM8_ESM.docx]

**Fig. S1 One *UAS* transgene in *30A> sona* and two *UAS* transgenes in *30A>GFP; sona* neither affect lethality by Sona overexpression nor lethal stage.**

Experiments were carried out three times at 25℃. Adult progenies from crosses between *30A-Gal4/CyO* and *CS* (64+100+76 from 3 experiments), *UAS-sona* (63+63+56 from 3 experiments), *UAS-GFP;* *UAS-sona* (38+55+37 from 3 experiments) were obtained. **a** No significant difference in survival rate between *30A>sona* and *30A>GFP; sona*. Data are presented as mean ± S.D. **b-d** Late stage pupae of *30A>+* (**b**), *30A>sona* (**c**) and *30A>GFP; sona* (**d**) flies are shown. In case of (**c**) and (**d**), dead pupae are shown.

**Fig. S2 Arr antibody test and expression of *arr* alleles.**

**a, b** Epitopes of the anti-Arr antibody is present in the intracellular domain of Arr. S2 cells were transfected with either *arr-HA* or *arr^m7^-HA*, and their CX and P100 fraction were used for analysis. Arr antibody recognized only overexpressed Arr-HA and endogenous Arr in both CX and P100. HA antibody recognized both Arr-HA and Arr^m7^-HA. **c, d** Arr^m7^-HA is not recognized by anti-Arr antibody in wing discs. Endogenous Arr was visualized by anti-Arr antibody (**c**). Arr^m7^-HA induced disorganization of posterior region and is recognized by anti-HA but not by -Arr antibody (**d**). **e, f** Arr-HA is recognized by both anti-HA (arrowhead in **e″**) and -Arr (arrowhead in **f″**) antibodies. **g** Knockdown of Arr by *arr RNAi* expression decreased the level of endogenous Arr but residual Arr is still present (**g‴**). Scale bar, 50μm.

**Fig. S3 Overexpression of Arr^m7^-HA induces developmental defects in wings and legs.**

**a, b** A control *nub-Gal4/+* wing is normal (**a**) and overexpression of *arm^m7^-HA* by *nub-Gal4* induced wing notching (**b**). **c, d** A control *ptc-Gal4/+* fly is normal (**c**) and overexpression of *arm^m7^-HA* by *ptc-Gal4* induced pupal lethality with no adult escaper (**d**). These pupae have defective wings and legs.

**Fig. S4 *arr^m7^* clones are not detected when the clones are generated in the first instar stage.**

Clonal analysis of *arr^m7^* generated at 1^st^ larvae stage by heat shock at 37℃ for 50 min. Twin spots (arrows) are detected but not *arr^m7^* clones. Scale bar, 50 μm.

**Fig. S5 *arr^2^* and *arr^m7^* clones show higher levels of both intra- and extra-cellular Wg.**

**a, b** MARCM analysis of *arr^2^* clones near the DV midline of wing discs. GFP regions indicate *arr^2^* clones (arrows). Levels of both extracellular and intracellular Wg are increased in *arr^2^* clones. **c, d** Clonal analysis of *arr^m7^* near the DV midline of wing discs. Regions without GFP mark *arr^2^* clones (arrows). Levels of both extracellular and intracellular Wg are increased in *arr^m7^* clones. Clones were generated at the 2^nd^ larvae stage by heat shock at 37℃ for 45-50 min. The arrows indicate homozygous clones. Scale bar, 20 μm.

**Fig. S6 The level of Sona was not changed by co-expression of Wg.**

S2 cells were transfected with *UAS-sona-HA*, *UAS-GFP-wg* and *UAS-arr* as well as *act-Gal4* plasmids. Co-expression of Wg actually reduced the amount of Sona compared to the ones transfected with only *sona*.

**Fig. S7 Sona did not affect Arr.**

**a, b** Arr banding patterns were not changed by Sona-Myc. Whole blots are shown for CX (**a**) and P100 (**b**) prepared from S2 cells transfected with constant amount of *arr-HA* plasmid and increasing amounts of *sona-Myc* plasmid. The amounts of full-length and active Sona were increased. **c, d** Sona affects neither transcription nor translation of *arr.* Compared to the control (**c**), *arr-lacZ; dpp>sona* wing discs (**d**) showed no change in the level of Arr and LacZ. *UAS-sona* was transiently expressed by temperature shift to 37℃ for 24 hrs just prior to dissection. Scale bar, 50 μm.
